# Supplementary material for: One reporter for in-cell activity profiling of majority of protein kinase oncogenes
Source: eLife. 2017 Feb 15;6:e21536. doi: 10.7554/eLife.21536 (PMC5310841; doi:10.7554/eLife.21536)
Supplement: Supplementary file 1. — (A) Commercial providers of RTK activity profiling; (B) Nucleotide sequences cloned into the promoterless pGL4.17 vector expressing firefly luciferase; (C) Expression vectors used in the study; (D) Antibodies used in the study; (E) Literature survey of anti-RTK activity of BCR-ABL TKIs; (F) Primers used for reporter construction. DOI: http://dx.doi.org/10.7554/eLife.21536.017 [file elife-21536-supp1.docx]

Supplementary Table A: Commercial providers of RTK activity profiling

(September 2016)

| *Company* | *Web* | *RTKs profiled (WT/mutant)* | *In-cell?* |
| --- | --- | --- | --- |
| BPS Bioscience | Bpsbioscience.com | 13/3 | No |
| MRC PPU | Ppu.mrc.ac.uk | 16/0 | No |
| Promega | Promega.com | 23/11 | No |
| Kinexus | Kinexus.ca | 36/2 | No |
| Luceome Biotech. | Luceome.com | 40/24 | No |
| Cisbio | Cisbio.com | 42/0 | No |
| Life Technologies | Lifetechnologies.com | 40/18 | No |
| Life Technologies | Lifetechnologies.com | 40/18 | No |
| Eurofins | Eurofins.com | 44/24 | No |
| ProQinase | Proquinase.com | 44/60 | No |
| Carna Biosciences | Carnabio.com | 46/39 | No |
| SignalChem | Signalchem.com | 49/146 | No |
| DiscoverX | Discoverx.com | 49/43 | No |
| Reaction Biology | Reactionbiology.com | 46/137 | No |
| DiscoverX | Discoverx.com | 30/0 | Yes |
| Carna Biosciences​ | Carnabio.com | 40/28 | Yes |
| Kinexus | Kinexus.ca | 28* | Yes |
| RnD Systems | Rndsystems.com | 49* | Yes |
| Cell Signaling | Cellsignal.com | 45* | Yes |
| Full Moon BioSys. | Fullmoonbio.com | 29* | Yes |
| PEPSCAN | Pepscan.com | 29** | Yes |

* The number of RTKs detected by specific antibodies in cell lysates

** The number of phosphorylated RTKs detected by γ-33P-ATP labelling

Supplementary Table B

| *Vector* | *Cloned sequence relative to TSS (bp)* |
| --- | --- |
| EGR1 | -1951/+161 (2112) |
| EGR2_1 | -2000/+150 (2150) |
| EGR2_2 | -945/+150 (1095) |
| EGR2_3 | -1601/+266 (1867) |
| EGR2_4 | -791/+266 (1057) |
| RGS1 | -936/+66 (1002) |
| NR4A2_1 | -1905/+133 (2038) |
| NR4A2_2 | -1131/+200 (1331) |
| DUSP6 | -1041/+288 (1329) |

Nucleotide sequences cloned into the promoterless pGL4.17 vector expressing firefly luciferase and analyzed for FGF2-mediated *trans*-activation at Fig. 1A. TSS, transcription start site.

Supplementary Table C: Expression vectors used in the study

| *Vector* | *Promoter* | *Insert* | *Backbone* | *Backbone source* |
| --- | --- | --- | --- | --- |
| pKrox24(2xD-E_inD)^Luc^ | 2 copies of D-E element in front of *EGR1* promoter fragment D, no other promoter elements | Firefly luciferase | pGL4.17 | Promega |
| pKrox24(2xD-E)^dTomato^ | 2 copies of D-E element, no other promoter elements | dTomato | pCLuc-Basic2 | New England Biolabs |
| pKrox24(2xD-E)^DsRed^ | 2 copies of D-E element, no other promoter elements | DsRed | pDsRed-Express-DR | Clontech |
| pKrox24(MapErk)^Luc^ | 5 copies of designed MapErk sequence in minimal promoter | Firefly luciferase | pGL4.26 | Promega |
| pKrox24(MapErk)^dTomato^ | 5 copies of designed MapErk sequence, no other promoter elements | dTomato | pCLuc-Basic2 | New England Biolabs |
| pKrox24(MapErk)^DsRed^ | 5 copies of designed MapErk sequence, no other promoter elements | DsRed | pDsRed-Express-DR | Clontech |
| pRL-TK | HSV-thymidine kinase | *Renilla* luciferase | pRL | Promega |
| RTK | CMV | Different RTKs | pcDNA3.1-V5/His | Invitrogen |
| ZAP70 | CMV | ZAP70 tyrosine kinase | pCMV6-Entry-Myc-DDK | Origene |
| SYK | CMV | SYK tyrosine kinase | pCMV6-Entry-Myc-DDK | Origene |
| TYK2 | CMV | TYK2 tyrosine kinase | [pCMV6-Entry-Myc-DDK](http://www.origene.com/destination_vector/PS100001.aspx) | Origene |
| ITK | CMV | ITK tyrosine kinase | [pCMV6-Entry-Myc-DDK](http://www.origene.com/destination_vector/PS100001.aspx) | Origene |
| FYN | CMV | FYN tyrosine kinase | pdEYFP-C1amp-YFP | ImaGenes |
| LYN | CMV | LYN tyrosine kinase | pDEST26-HIS6 | ImaGenes |
| BLK | CMV | BLK tyrosine kinase | pdEYFP-C1amp-YFP | ImaGenes |
| FYN | CMV | FYN tyrosine kinase | pdEYFP-C1amp-YFP | ImaGenes |
| YES | CMV | YES tyrosine kinase | pdEYFP-C1amp-YFP | ImaGenes |
| LCK | CMV | LCK tyrosine kinase | pDEST26-HIS6 | ImaGenes |
| FGR | CMV | FGR tyrosine kinase | pCMV6-Entry-Myc-DDK | Origene |
| TEC | CMV | TEC tyrosine kinase | pCMV6-Entry-Myc-DDK | Origene |
| ABL | CMV | ABL tyrosine kinase | pCR3.1-FLAG | Invitrogen |
| BCR-ABL p190 | CMV | BCR-ABL, p190 | pCR3.1-FLAG | Invitrogen |
| BCR-ABL p210 | CMV | BCR-ABL, p210 | pCR3.1-FLAG | Invitrogen |
| C-RAF^CAAX^ | CMV | C-RAF-CAAX | pCMV | Clontech |
| B-RAF^V600E^ | CMV | B-RAF-V600E | pCMV6-Entry-Myc-DDK | Origene |
| RAS | CMV | RAS | pCMV | Clontech |
| RAS^V12^ | CMV | RAS-G12V | pCMV | Clontech |

Supplementary Table D: Antibodies used in the study

| *Kinase* | *Antibody ^pY^* | *Catalog #* | *Manufacturer* | *Total protein Ab* | *RRID* |
| --- | --- | --- | --- | --- | --- |
| ABL | ABL^Y412^ | 2865 | Cell Signaling | FLAG | AB_331381 |
| ALK | ALK^Y1096^ | 6962 | Cell Signaling | ALK | AB_10828357 |
| BLK | 4G10^panY^ | 05-321 | Millipore | GFP | AB_309678 |
| LTK | ALK ^Y1278/Y1282/Y1283^ | 3983 | Cell Signaling | V5 | AB_10545761 |
| AXL | AXL^Y702^ | 5724 | Cell Signaling | V5 | AB_10548763 |
| DDR1 | 4G10^panY^ | 05-321 | Millipore | V5 | AB_309678 |
| DDR2 | 4G10^panY^ | 05-321 | Millipore | V5 | AB_309678 |
| EGFR | EGFR^Y992^ | 2235 | Cell Signaling | V5 | AB_331709 |
| ERBB2 | ERBB2^Y877^ | 2241 | Cell Signaling | V5 | AB_2099407 |
| ERBB4 | ERBB4^Y984^ | 3790 | Cell Signaling | V5 | AB_2099879 |
| FGFR1 | FGFR^Y653/Y654^ | 3476 | Cell Signaling | V5 | AB_331369 |
| FGFR2 | FGFR^Y653/Y654^ | 3476 | Cell Signaling | V5 | AB_331369 |
| FGFR3 | FGFR^Y653/Y654^ | 3471 | Cell Signaling | FGFR3 | AB_331072 |
| FGFR4 | FGFR^Y653/Y654^ | 3471 | Cell Signaling | FGFR4 | AB_331072 |
| FGR | 4G10^panY^ | 05-321 | Millipore | GFP | AB_309678 |
| IGF1R | IGF1R^Y1135^ | 3918 | Cell Signaling | V5 | AB_10548764 |
| INSR | INSR^Y1345^ | 3026 | Cell Signaling | V5 | AB_2127116 |
| ITK | 4G10^panY^ | 05-321 | Millipore | FLAG | AB_309678 |
| MET | MET^Y1234/Y1235^ | 3077 | Cell Signaling | V5 | AB_2143884 |
| RON | MET^Y1234/Y1235^, MET^Y1003^ | 3077, 3135 | Cell Signaling | V5 | AB_2143884, AB_2285325 |
| CSF1R | CSF1R^Y699^ | 12251 | Cell Signaling | V5 | AB_2636867 |
| FLT3 | FLT3^Y842^ | 4577 | Cell Signaling | V5 | AB_916078 |
| KIT | KIT^Y703^ | 3073 | Cell Signaling | V5 | AB_1147635 |
| PDGFRA | PDGFRA^Y762^ | 12022 | Cell Signaling | V5 | AB_2636868 |
| PDGFRB | PDGFRA^Y849^/B^Y857^ | 3170 | Cell Signaling | V5 | AB_2162348 |
| RET | RET^Y905^ | 3221 | Cell Signaling | RET | AB_2179887 |
| SYK | 4G10^panY^ | 05-321 | Millipore | FLAG | AB_309678 |
| TEC | 4G10^panY^ | 05-321 | Millipore | FLAG | AB_309678 |
| TEK | TIE 2^Y992^ | 4221 | Cell Signaling | V5 | AB_2203198 |
| TRKA | TRKA^Y674/Y675^/B^Y706/Y707^ | 4621 | Cell Signaling | V5 | AB_916186 |
| TRKB | TRKA^Y674/Y675^/B^Y706/Y707^ | 4621 | Cell Signaling | V5 | AB_916186 |
| TRKC | TRKA^Y674/Y675^/B^Y706/Y707^ | 4621 | Cell Signaling | V5 | AB_916186 |
| TYK2 | 4G10^panY^ | 05-321 | Millipore | FLAG | AB_309678 |
| VEGFR2 | VEGFR2^Y1059^ | 3817 | Cell Signaling | V5 | AB_2132351 |
| VEGFR3 | VEGFR3^Y1230/Y1231^ | CY1115 | Cell Applications | V5 | AB_2636869 |
| YES | 4G10^panY^ | 05-321 | Millipore | GFP | AB_309678 |
| ZAP70 | 4G10^panY^ | 05-321 | Millipore | FLAG | AB_309678 |
| ERK | pERK^T202/Y204^ | 4376 | Cell Signaling | ERK | AB_331772 |
| STAT1 | pSTAT1^Y701^ | 9167 | Cell Signaling | STAT1 | AB_561284 |
| *Other Abs* |  | *Catalog#* | *Manufacturer* |  |  |
| V5 | - | R960-25 | Invitrogen | - | AB_2556564 |
| ALK | - | 3333 | Cell Signaling | - | AB_836862 |
| FGFR3 | - | sc-123 | Santa Cruz | - | AB_631511 |
| FGFR4 | - | 2894 | Cell Signaling | - | AB_2293993 |
| RET | - | 3223 | Cell Signaling | - | AB_2238465 |
| FLAG | - | F1804 | Sigma-Aldrich | - | AB_262044 |
| GFP | - | 2555 | Cell Signaling | - | AB_10692764 |
| LCK | - | 2752 | Cell Signaling | - | AB_2234649 |
| LYN | - | 2732 | Cell Signaling | - | AB_10694080 |
| B-RAF | - | sc-5284 | Santa Cruz | - | AB_626760 |
| C-RAF | - | 610151 | BD Bioscence | - | AB_397552 |
| RAS | - | 610001 | BD Bioscence | - | AB_397424 |
| EGR1 | - | 4154 | Cell Signaling | - | AB_2097035 |
| ERK | - | 9102 | Cell Signaling | - | AB_330744 |
| RFP | - | 600-401-379 | Rockland | - | AB_2209751 |
| Actin | - | 3700 | Cell Signaling | - | AB_2242334 |
| STAT1 | - | 9172 | Cell Signaling | - | AB_10693929 |
| GST | - | G1160 | Sigma-Aldrich | - | AB_259845 |

Total levels of RTKs were determined by V5 antibody; ALK, FGFR3, FGFR4 and RET expression was determined with specific antibody. RFP antibody was used for immunoblot detection of dTomato and dsRED. Phosphorylated (p) LTK and RON were determined with pALK and pMET antibodies, respectively. 4G10, pan-pTyr antibody.

Supplementary Table E: Literature survey of anti-RTK activity of BCR-ABL TKIs

| *RTK* | *Ponatinib* | *Imatinib* | *Osimertinib* | *Dasatinib* | *Bosutinib* | *Nilotinib* | *References* |
| --- | --- | --- | --- | --- | --- | --- | --- |
| ALK | N¶ | N¶ | Y¶ | N*¶* | Y¶ Y*** | N¶* | 1-7 |
| LTK | N¶ | N¶ | - | N*¶* | - | N¶ | 2, 3, 5 |
| AXL | N¶ | N¶ | - | N*¶* | Y¶* | N¶ | 2, 3, 5, 8 |
| DDR1 | Y¶* | Y¶* | - | Y¶* | Y¶ | Y¶* | 2, 4, 9-13 |
| DDR2 | Y¶ | Y¶* | - | Y¶* | Y¶ | Y¶* | 2-5, 7, 9, 12-14 |
| EGFR | N¶* | N¶ | Y¶* | Y¶* | Y¶ | N¶ | 1, 2, 5, 7, 15-18 |
| ERBB2 | N¶ | N¶ | Y¶* | Y¶ | N*¶* | N¶ | 1, 2, 5, 19 |
| ERBB4 | Y¶ | Y¶ | Y¶ | Y¶ | Y¶ | Y¶ | 1, 2, 7 |
| FGFR1 | Y¶* | N¶ | Y¶ | Y¶ | Y¶ | N¶* | 1-5, 7, 20 |
| FGFR2 | Y¶* | N¶ | - | Y¶ | Y¶ | N¶* | 2, 4, 5, 7, 20 |
| FGFR3 | Y¶* | N¶ | - | Y¶ | Y¶ | N¶* | 2-5, 7, 20 |
| FGFR4 | Y¶* | N¶ | - | N*¶* | N*¶* | N¶* | 2-5, 7, 21 |
| IGF1R | N¶ | N¶ | Y¶ | N*¶* Y* | N*¶* | N¶* | 1-5, 7, 22, 23 |
| INSR | N¶ | N¶ | Y¶ | N*¶* | N*¶* | N¶* | 1, 2, 4, 5 |
| MET | N¶ | N¶ | Y* | N*¶* Y* | Y¶ | N¶* | 1-5, 7, 24-26 |
| RON | N¶ | N¶ | - | N¶ | Y¶ | N¶ | 2, 3, 5, 7 |
| CSF1R | Y¶ | Y¶* | - | Y¶ * | Y¶ | Y¶* | 2-5, 9, 27-30 |
| FLT3 | Y¶* | Y¶ N* | Y¶ | N*¶* | Y¶ | Y¶ N* | 1, 2, 5, 7, 31-33 |
| KIT | Y¶* | Y¶* | - | Y¶* | Y¶ | Y¶* | 2, 4, 5, 7, 9, 31, 32, 34-37 |
| PDGFRA | Y¶* | Y¶* | - | Y¶* | N¶ | Y¶* | 2-5, 7, 9, 20, 31, 37-39 |
| PDGFRB | Y¶* | Y¶* | - | Y¶* | Y¶ | Y¶* | 2-5, 9, 31, 37, 40, 41 |
| RET | Y¶* | Y* N¶ | - | Y¶ | Y¶ | Y¶ N* | 2-5, 7, 42, 43 |
| TEK | Y¶ | N¶ | - | N¶ | Y¶ | N¶ | 2, 3, 5, 7 |
| TRKA | Y¶ | N¶* | - | Y¶ | Y¶ | Y¶ | 2, 3, 5, 7, 44 |
| TRKB | Y¶ | N¶ | N¶ | N¶ | Y¶ | Y¶ | 1, 2, 5, 7 |
| TRKC | Y¶ | N¶ | - | Y¶ | - | Y¶ | 2, 5 |
| VEGFR2 | Y¶ | N¶ | - | Y¶ | N¶ | Y¶ | 2, 5, 7 |
| VEGFR3 | Y¶ | N¶ | Y¶ | Y¶ | Y¶ | N¶ | 1, 2, 5, 7 |

* in-cell assay

¶ cell-free assay

References

1. Cross, D.A.E. et al. AZD9291, an Irreversible EGFR TKI, Overcomes T790M-Mediated Resistance to EGFR Inhibitors in Lung Cancer. *Cancer Discovery* **4**, 1046-1061 (2014).

2. Kitagawa, D. et al. Activity-based kinase profiling of approved tyrosine kinase inhibitors. *Genes to Cells* **18**, 110-122 (2013).

3. Liu, X., Kung, A., Malinoski, B., Prakash, G.K.S. & Zhang, C. Development of Alkyne-Containing Pyrazolopyrimidines To Overcome Drug Resistance of Bcr-Abl Kinase. *Journal of Medicinal Chemistry* **58**, 9228-9237 (2015).

4. Manley, P.W. et al. Extended kinase profile and properties of the protein kinase inhibitor nilotinib. *Biochimica Et Biophysica Acta-Proteins and Proteomics* **1804**, 445-453 (2010).

5. O'Hare, T. et al. AP24534, a Pan-BCR-ABL Inhibitor for Chronic Myeloid Leukemia, Potently Inhibits the T315I Mutant and Overcomes Mutation-Based Resistance. *Cancer Cell* **16**, 401-412 (2009).

6. Puttini, M. et al. In vitro and in vivo activity of SKI-606, a novel Src-Abl inhibitor, against imatinib-resistant Bcr-Abl(+) neoplastic cells. *Cancer Research* **66**, 11314-11322 (2006).

7. Rix, L.L.R. et al. Global target profile of the kinase inhibitor bosutinib in primary chronic myeloid leukemia cells. *Leukemia* **23**, 477-485 (2009).

8. Zhang, Y.X. et al. AXL is a potential target for therapeutic intervention in breast cancer progression. *Cancer Research* **68**, 1905-1915 (2008).

9. Manley, P.W. et al. Structural resemblances and comparisons of the relative pharmacological properties of imatinib and nilotinib. *Bioorganic & Medicinal Chemistry* **18**, 6977-6986 (2010).

10. Rix, U. et al. Chemical proteomic profiles of the BCR-ABL inhibitors imatinib, nilotinib, and dasatinib, reveal novel kinase and nonkinase targets. *Blood* **110**, 4055-4063 (2007).

11. Bantscheff, M. et al. Quantitative chemical proteomics reveals mechanisms of action of clinical ABL kinase inhibitors. *Nature Biotechnology* **25**, 1035-1044 (2007).

12. Canning, P. et al. Structural Mechanisms Determining Inhibition of the Collagen Receptor DDR1 by Selective and Multi-Targeted Type II Kinase Inhibitors. *Journal of Molecular Biology* **426**, 2457-2470 (2014).

13. Day, E. et al. Inhibition of collagen-induced discoidin domain receptor 1 and 2 activation by imatinib, nilotinib and dasatinib. *European Journal of Pharmacology* **599**, 44-53 (2008).

14. Terai, H. et al. Characterization of DDR2 Inhibitors for the Treatment of DDR2 Mutated Nonsmall Cell Lung Cancer. *Acs Chemical Biology* **10**, 2687-2696 (2015).

15. Aggerholm-Pedersen, N. et al. Dasatinib and Doxorubicin Treatment of Sarcoma Initiating Cells: A Possible New Treatment Strategy. *Stem Cells International* (2016).

16. Lin, Y.C. et al. Degradation of Epidermal Growth Factor Receptor Mediates Dasatinib-Induced Apoptosis in Head and Neck Squamous Cell Carcinoma Cells. *Neoplasia* **14**, 463-475 (2012).

17. Nautiyal, J., Majumder, P., Patel, B.B., Lee, F.Y. & Majumdar, A.P.N. Src inhibitor dasatinib inhibits growth of breast cancer cells by modulating EGFR signaling. *Cancer Letters* **283**, 143-151 (2009).

18. Lee, H.J. et al. Drug Resistance via Feedback Activation of Stat3 in Oncogene-Addicted Cancer Cells. *Cancer Cell* **26**, 207-221 (2014).

19. Shen, X.K. et al. A systematic analysis of the resistance and sensitivity of HER2(YVMA) receptor tyrosine kinase mutant to tyrosine kinase inhibitors in HER2-positive lung cancer. *Journal of Receptors and Signal Transduction* **36**, 89-97 (2016).

20. Gozgit, J.M. et al. Ponatinib (AP24534), a Multitargeted Pan-FGFR Inhibitor with Activity in Multiple FGFR-Amplified or Mutated Cancer Models. *Molecular Cancer Therapeutics* **11**, 690-699 (2012).

21. Li, S.Q. et al. Targeting Wild-Type and Mutationally Activated FGFR4 in Rhabdomyosarcoma with the Inhibitor Ponatinib (AP24534). *Plos One* **8** (2013).

22. Dayyani, F. et al. Combined Inhibition of IGF-1R/IR and Src Family Kinases Enhances Antitumor Effects in Prostate Cancer by Decreasing Activated Survival Pathways. *Plos One* **7** (2012).

23. Min, H.Y. et al. Targeting the insulin-like growth factor receptor/Insulin receptor and Src signaling network for the treatment of non-small cell lung cancer. *Cancer Research* **74** (2014).

24. Nehoff, H., Parayath, N.N., McConnell, M.J., Taurin, S. & Greish, K. A combination of tyrosine kinase inhibitors, crizotinib and dasatinib for the treatment of glioblastoma multiforme. *Oncotarget* **6**, 37948-37964 (2015).

25. Marley, K., Gullaba, J., Seguin, B., Gelberg, H.B. & Helfand, S.C. Dasatinib Modulates Invasive and Migratory Properties of Canine Osteosarcoma and has Therapeutic Potential in Affected Dogs. *Translational Oncology* **8**, 231-238 (2015).

26. Liu, S.Y. et al. Targeting tyrosine-kinases and estrogen receptor abrogates resistance to endocrine therapy in breast cancer. *Oncotarget* **5**, 9049-9064 (2014).

27. Chase, A. et al. Imatinib sensitivity as a consequence of a CSF1R-Y571D mutation and CSF1/CSF1R signaling abnormalities in the cell line GDM1. *Leukemia* **23**, 358-364 (2009).

28. Dewar, A.L. et al. Macrophage colony-stimulating factor receptor c-fms is a novel target of imatinib. *Blood* **105**, 3127-3132 (2005).

29. Uitdehaag, J.C.M. et al. Multidimensional Profiling of CSF1R Screening Hits and Inhibitors: Assessing Cellular Activity, Target Residence Time, and Selectivity in a Higher Throughput Way. *Journal of Biomolecular Screening* **16**, 1007-1017 (2011).

30. Uitdehaag, J.C.M. et al. A guide to picking the most selective kinase inhibitor tool compounds for pharmacological validation of drug targets. *British Journal of Pharmacology* **166**, 858-876 (2012).

31. Buchdunger, E. et al. Abl protein-tyrosine kinase inhibitor STI571 inhibits in vitro signal transduction mediated by c-Kit and platelet-derived growth factor receptors. *Journal of Pharmacology and Experimental Therapeutics* **295**, 139-145 (2000).

32. Gozgit, J.M. et al. Potent Activity of Ponatinib (AP24534) in Models of FLT3-Driven Acute Myeloid Leukemia and Other Hematologic Malignancies. *Molecular Cancer Therapeutics* **10**, 1028-1035 (2011).

33. Mashkani, B., Tanipour, M.H., Saadatmandzadeh, M., Ashman, L.K. & Griffith, R. FMS-like tyrosine kinase 3 (FLT3) inhibitors: Molecular docking and experimental studies. *European Journal of Pharmacology* **776**, 156-166 (2016).

34. Galanis, A. & Levis, M. Inhibition of c-Kit by tyrosine kinase inhibitors. *Haematologica* **100**, E77-E79 (2015).

35. Heinrich, M.C. et al. Inhibition of c-kit receptor tyrosine kinase activity by STI 571, a selective tyrosine kinase inhibitor. *Blood* **96**, 925-932 (2000).

36. Dos Santos, C. et al. The Src and c-Kit kinase inhibitor dasatinib enhances p53-mediated targeting of human acute myeloid leukemia stem cells by chemotherapeutic agents. *Blood* **122**, 1900-1913 (2013).

37. Weisberg, E. et al. Characterization of AMN107, a selective inhibitor of native and mutant Bcr-Abi (vol 7, pg 129, 2005). *Cancer Cell* **7**, 399-399 (2005).

38. Bai, Y. et al. Phosphoproteomics Identifies Driver Tyrosine Kinases in Sarcoma Cell Lines and Tumors. *Cancer Research* **72**, 2501-2511 (2012).

39. Truffaux, N. et al. Preclinical evaluation of dasatinib alone and in combination with cabozantinib for the treatment of diffuse intrinsic pontine glioma. *Neuro-Oncology* **17**, 953-964 (2015).

40. Arts, F.A. et al. PDGFRB mutants found in patients with familial infantile myofibromatosis or overgrowth syndrome are oncogenic and sensitive to imatinib. *Oncogene* **35**, 3239-3248 (2016).

41. Dickerson, E.B. et al. Imatinib and Dasatinib Inhibit Hemangiosarcoma and Implicate PDGFR-beta and Src in Tumor Growth. *Translational Oncology* **6**, 158-168 (2013).

42. de Groot, J.W.B. et al. Cellular effects of imatinib on medullary thyroid cancer cells, harboring multiple endocrine neoplasia Type 2A and 2B associated RET mutations. *Surgery* **139**, 806-814 (2006).

43. De Falco, V. et al. Ponatinib (AP24534) Is a Novel Potent Inhibitor of Oncogenic RET Mutants Associated With Thyroid Cancer. *Journal of Clinical Endocrinology & Metabolism* **98**, E811-E819 (2013).

44. Koch, A. et al. Inhibition of Abl tyrosine kinase enhances nerve growth factor-mediated signaling in Bcr-Abl transformed cells via the alteration of signaling complex and the receptor turnover. *Oncogene* **27**, 4678-4689 (2008).

Supplementary Table F: Primers used for reporter construction

| *Name of sequence* | *Primer*  *(restriction site in grey)* | | *Restriction site* | |
| --- | --- | --- | --- | --- |
| hEGR1-A | forward | *GGTACC*TCGGTAGACAGTGGGAGTGA | Kpn I |  |
|  | reverse | *GATATC*GGAACACTGAGAAGCGTGCAGG | EcoR V |  |
| hEGR1-B | forward | *GGTACC*AAAAAACAGCACCTCCTCTGGAT | Kpn I |  |
|  | reverse | *GATATC*GGAACACTGAGAAGCGTGCAGG | EcoR V |  |
| hEGR1-C | forward | *GGTACC*AGGAGGCGGCGGAAGAG | Kpn I |  |
|  | reverse | *GATATC*GGAACACTGAGAAGCGTGCAGG | EcoR V |  |
| hEGR1-D | forward | *GGTACC*AAAGACACCGTGCCATAGATCGA | Kpn I |  |
|  | reverse | *GATATC*GGAACACTGAGAAGCGTGCAGG | EcoR V |  |
| hEGR1-E | forward | *GGTACC*AACAACCCTTATTTGGGCAGCA | Kpn I |  |
|  | reverse | *GATATC*GGAACACTGAGAAGCGTGCAGG | EcoR V |  |
| hEGR1-F | forward | *GGTACC*CTAGAGCTCTAGGCTTCCC | Kpn I |  |
|  | reverse | *GATATC*GGAACACTGAGAAGCGTGCAGG | EcoR V |  |
| hEGR1-D2 | forward | *GGTACC*ACGCCTAGGAGCCGCCTGA | Kpn I |  |
|  | reverse | *GATATC*GGAACACTGAGAAGCGTGCAGG | EcoR V |  |
| hEGR1-D3 | forward | *GGTACC*GCTCCCGGCTTGGAACCA | Kpn I |  |
|  | reverse | *GATATC*GGAACACTGAGAAGCGTGCAGG | EcoR V |  |
| hEGR1-D4 | forward | *GGTACC*AAAGACACCGTGCCATAGATCGA | Kpn I |  |
|  | reverse | *GATATC*GGCTCCCCAAGTTCTGCGCG | EcoR V |  |
| hEGR1-D5 | forward | *GGTACC*AAAGACACCGTGCCATAGATCGA | Kpn I |  |
|  | reverse | *GATATC*CGGTCCTGCGGCGGCGGAAG | EcoR V |  |
| hEGR2_1 | forward | *GGTACC*CACATCCTTCAGATCTCTGCTTA | Kpn I |  |
|  | reverse | *GATATC*GGAGGAGGATGCCAGTAGAA | EcoR V |  |
| hEGR2_2 | forward | *GGTACC*CCTGATACATCTTGGAGT | Kpn I |  |
|  | reverse | *GATATC*GGAGGAGGATGCCAGTAGAA | EcoR V |  |
| hEGR2_3 | forward | *GGTACC*GCAACCTGCACAAACGACCATGAAT | Kpn I |  |
|  | reverse | *GATATC*GGTTGGACTGAGCCTGGGATGG | EcoR V |  |
| hEGR2_4 | forward | *GGTACC*GGTGTCTCCGGCTGAGGATTT | Kpn I |  |
|  | reverse | *GATATC*GGTTGGACTGAGCCTGGGATGG | EcoR V |  |
| RGS1 | forward | *CTCGAG*CTGTTATCTCTCCAGAGATACTGCC | XhoI |  |
|  | reverse | *AGATCT*GGTGCTCTTAGCAAATATGCGCTAGTC | BglII |  |
| NR4A2_1 | forward | *GGTACC*ACCGAGCTCATGCTAATATGCT | Kpn I |  |
|  | reverse | *GATATC*AAGGGAACCCGGACACCT | EcoR V |  |
| NR4A2_2 | forward | *GGTACC*CACACCTTACGCTTTGCGGA | Kpn I |  |
|  | reverse | *GATATC*CTGCCGAAGTGCAGTTCCCTCTG | EcoR V |  |
| DUSP6 | forward | *GGTACC*GTGTTCACGGTAGGCGCAAA | Kpn I |  |
|  | reverse | *GATATC*CTTCTTTAGGCGGTGTGTGGCA | EcoR V |  |
| element D-E | forward | *GGTACC*AAAGACACCGTGCCATAGATCGA | Kpn I |  |
|  | reverse | *GGTACC*GGATCCTTCCTGCTCCTTATATGG | Kpn I |  |
| element D-D2 | forward | *GGTACC*AAAGACACCGTGCCATAGATCGA | Kpn I |  |
|  | reverse | *GGTACC*GAACTAGGCTGGGGAAGCCC | Kpn I |  |
| BCR-ABLp190 | forward | *GATATC*TGGTGGACCCGGTGGGCTT | EcoRV |  |
|  | reverse | *GCGGCCGC*CTACCTCTGCACTATG | NotI |  |
| BCR-ABLp210 | forward | *GATATC*TGGTGGACCCGGTGGGCTT | EcoRV |  |
|  | reverse | *GCGGCCGC*CTACCTCTGCACTATG | NotI |  |
